# Winter Nursing Engagements

**Published:** 1888-03-03

**Authors:** 


### THE NURSING MIRROR.

Communications for this column should be addressed The Mirror, care of the Editor, 38, Old Jewry, London, E.C.

### WINTER NURSING ENGAGEMENTS.

A "Private Nurse" requires answers to the following questions:

1. Where is a nurse to apply who is desirous of joining the English Nursing Staff at Nice or any other places on the Riviera during the winter season? and is it not advisable to make such application early in the spring?

2. Where is a nurse to apply who wishes to go out to India to nurse in the Government or military hospitals?

3. If a nurse is doing private nursing for herself, would it be necessary for her to return to hospital for a year in order to obtain a certificate?

